# Advances in selenium supplementation: From selenium-enriched yeast to potential selenium-enriched insects, and selenium nanoparticles

**DOI:** 10.1016/j.aninu.2023.05.002

**Published:** 2023-05-20

**Authors:** Luca Ferrari, Donata M.I.R. Cattaneo, Rossella Abbate, Michele Manoni, Matteo Ottoboni, Alice Luciano, Christoph von Holst, Luciano Pinotti

**Affiliations:** a Department of Veterinary Medicine and Animal Sciences (DIVAS), Università degli Studi di Milano, 26900 Lodi, Italy; b European Commission, Joint Research Centre (JRC), 2440 Geel, Belgium; c CRC I-WE (Coordinating Research Centre: Innovation for Well-Being and Environment), Università degli Studi di Milano, 20133 Milan, Italy

**Keywords:** Selenium, Selenium-enriched yeast, Selenium nanoparticle, Selenium-enriched insect, Speciation analysis

## Abstract

Selenium (Se) is an essential micronutrient that plays an important role in animal and human development and physiological homoeostasis. This review surveys the role of Se in the environment, plants and animal bodies, and discusses data on Se biofortification with different sources of supplementation, from inorganic to organic forms, with special focus on Se-enriched yeast (Se-yeast). Although Se-yeast remains one of the main sources of organic Se, other emerging and innovative sources are reviewed, such as Se-enriched insects and Se-nanoparticles and their potential use in animal nutrition. Se-enriched insects are discussed as an option for supplying Se in organic form to livestock diets. Se-nanoparticles are also discussed, as they represent a more biocompatible and less toxic source of inorganic Se for animal organisms, compared to selenite and selenate. We also provide up to date information on the legal framework in the EU, USA, and Canada of Se that is contained in feed additives. From the scientific evidence available in the literature, it can be concluded that among the inorganic forms, sodium selenite is still one of the main options, whereas Se-yeast remains the primary organic form. However, other potential sources such as Se-enriched insects and Se-nanoparticles are being investigated as they could potentially combine a high bioavailability and reduced Se emissions in the environment.

## 1 Introduction

Selenium (Se) is an important trace element, essential for both humans and animals (Constantinescu-Aruxandei et al., 2018). Selenium is involved in various biochemical reactions and adequate intake is fundamental for organisms to function (D'Amato et al., 2020). For example, Se acts as a cofactor of several enzyme complexes such as glutathione peroxidase (Schiavon et al., 2020) or thioredoxin reductase (Zoidis et al., 2018), all enzymes that protect the body's cells from damage due to radical processes (Newberne and Suphakarn, 1983; Thompson and Scott, 1969).

Similarly, Se is a cofactor of iodothyronine deiodinase, an important enzyme involved in the metabolism of thyroid hormones (Adadi et al., 2019). The recommended daily allowance (RDA) of Se in a properly functioning organism is 55 mg for both women and men (Bodnar et al., 2016) and, when this intake drops under minimum daily intake, acute or chronic diseases may arise due to Se deficiency. A deficiency in the diet can therefore lead to serious consequences such as infertility, cardiovascular disease, and cancer due to oxidative stress (Broadley et al., 2006).

Beneficial effects of an adequate intake have been found not only in humans but also in animals. For example, in commercial poultry production, regulating the antioxidant defence system of animals through optimal dietary levels of vitamin E, Se, and carotenoids, helps maintain the productive and reproductive performance of poultry (Surai et al., 2019). In addition, maximum protection is provided against the oxidative stress to which these animals are exposed (Surai et al., 2019).

As in humans, Se deficiency in the diet can cause several problems to livestock. In cattle, Se deficiency can result in reduced fertility, placental retention and increased incidence of mastitis and metritis (Baldi et al., 2000; Mehdi and Dufrasne, 2016), while it can cause mulberry heart disease in pigs (Oropeza–Moe et al., 2015) and cardiac injury in chickens (Liu et al., 2020).

Compared to other minerals, such as copper (Cu), iron (Fe) and zinc (Zn), Se has a narrow range between deficiency and toxicity (Kobayashi et al., 2018; Mocchegiani et al., 2013; Scheiber et al., 2013). In fact, Se can induce negative effects when overexposure takes place. In this case, Se has the opposite effects: from an antioxidant, it becomes pro-oxidant in the body's cells (Gu and Gao, 2022).

Due to the significant impacts of Se deficiency, strategies based on fertilisation (using Se fertilisers) can ensure an adequate supply of Se for both crops intended for direct human consumption and/or crops for livestock. Biofortification with Se fertilizers in crops as pastures or fodder is an effective way of increasing human and/or animal Se intake (Broadley et al., 2006; White et al., 2004). A recent advance in this research field is the biofortification of feed/food ingredients. Currently, Se-enriched yeast (Se-yeast) is the main source of organic Se, however, a recent progress in this area has suggested that insects could be an alternative in animal nutrition, as well as using Se in the form of nanoparticles.

In the present work, we present an overview of Se in the environment, in plants and in animal organisms, as well as the different well-established sources of supplementation. Potential biofortification strategies, such as Se-enriched insects and Se nanotechnology, are also presented. We also provide information on the legal framework of Se containing feed additives in the EU, USA, and Canada.

## 2 Selenium in the feed/food chain

**Fig. 1:**
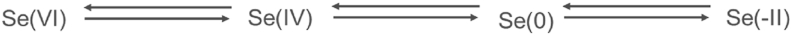
Interconversion mechanism of the 4 selenium (Se) oxidation states.

It is generally recognised that the mobility, toxicity, and bioavailability of trace elements, including Se, depends on their chemical form (Ochsenkühn–Petropoulou et al., 2006). Selenium, in particular, exists in several forms in the environment (Fig. 1), but its most abundant forms are selenite (SeO_3_^2−^, Se(IV)) and selenate (SeO_4_^2−^, Se(VI)) (Kumar and Prasad, 2021), where Se(IV) is considered more toxic than Se(VI) (Arshad et al., 2021). Plant-based products are the route by which Se enters the food chain, since the intake through drinking water is generally minimal (Hossain et al., 2021). However, disparity in Se content between foods, depending on the Se content of the soil, must be taken into account (Cattaneo et al., 2008). Therefore, it is important to monitor the speciation of Se, the exposure level, as well as the mode of action of the different species on organisms.

**Fig. 2:**
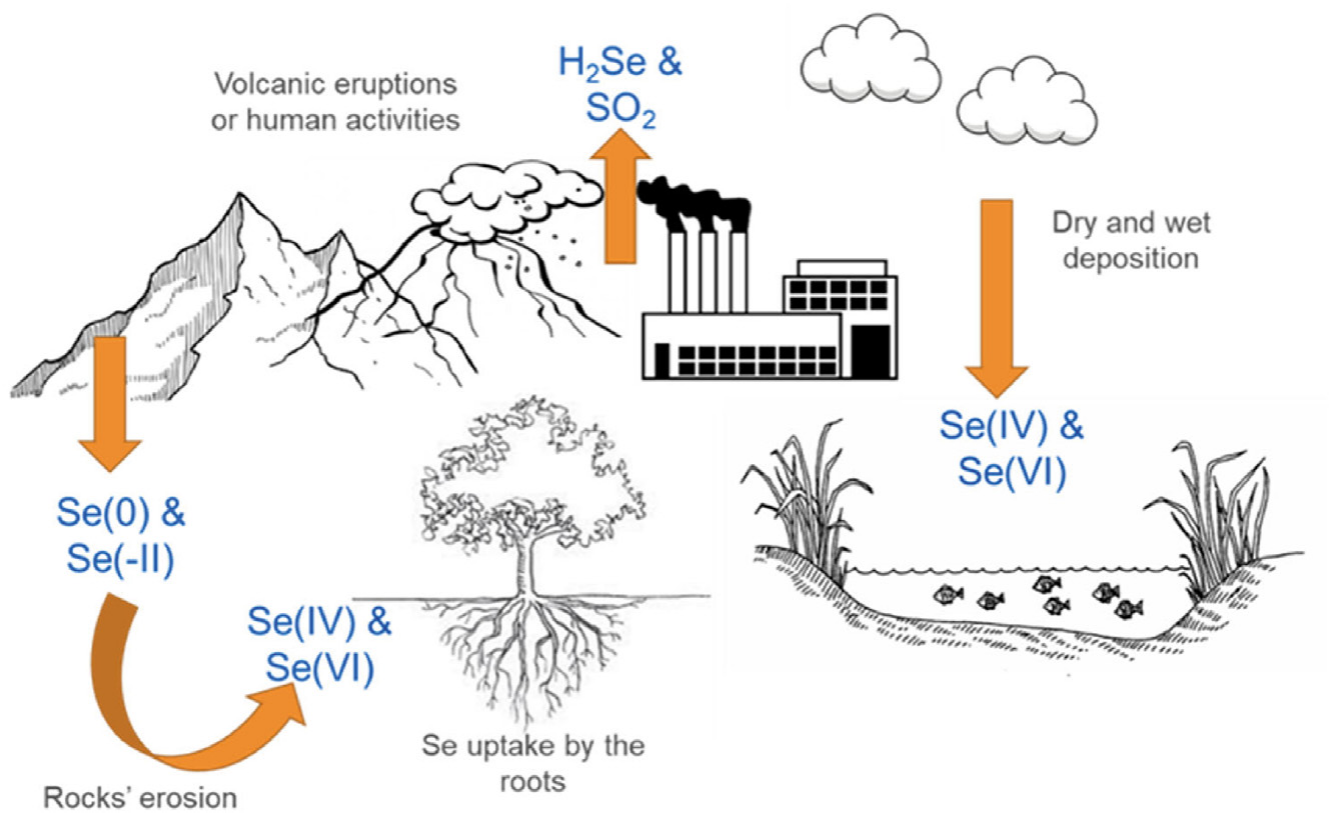
Selenium (Se) natural cycle. Selenium is released into the atmosphere thanks to volcanic eruptions or fossil combustions, while the release into the soil occurs by solubilization of minerals in rocks. Once released, it is absorbed by plants and aquatic organisms entering the food chain.

Selenium is found in soil and water. Its presence is linked to the precipitation of Se species in the atmosphere or to the erosion of rocks containing selenites and/or selenides (Mehdi et al., 2013). This means that in both water and soil, Se is found mainly as inorganic salts as well as in elemental form (Fig. 2). Selenite (SeO_3_^2−^) and selenate (SeO_4_^2−^) are common in most soils and these anionic forms of Se are highly soluble, mobile, bioavailable, and potentially toxic.

The presence of Se in water and soil affects its uptake by plants. In particular, Se uptake is influenced by the concentration of Se in soil, although Se speciation, the concentration of competing anions (like SO_4_^2−^), pH, and redox conditions have their importance (Puccinelli et al., 2017). The average Se content in soils, calculated worldwide, is reported to be 0.4 mg Se/kg, although concentrations around 1,200 mg Se/kg can be reached in seleniferous soil in countries as USA, Canada, Colombia, UK, China, and Russia (Tan et al., 2016). Plants absorb these forms from their roots and absorption takes place via the same mechanisms and transporters of sulphur (S), due to the similarity between these two elements (Puccinelli et al., 2017; White et al., 2004). While Se is essential for many animals and bacteria, as well as the green algae *Chlamydomonas reinhardtii*, there is still no evidence that Se is an essential element for higher plants as well (Fu et al., 2002). However, while there is no proof of essentiality, there have been reports of beneficial effects of Se on plant growth as well as there is increasing evidence that Se can also protect plants from biotic stresses (Pilon-Smits et al., 2009). Selenium is chemically similar to S and, once absorbed as SeO_3_^2−^ and SeO_4_^2−^, is reduced to selenide (Se^2−^) and assimilated by plants into amino acids including selenocysteine (SeCys) and selenomethionine (SeMet). Assimilation via the S assimilation pathway represents a mechanism of the plant to reduce Se toxicity (Pilon-Smits et al., 2009): in this way, Se becomes less toxic but also more bioavailable along the food chain.

### 2.1 Selenium in animals: an overview

Selenoproteins, such as the glutathione peroxidase family and thioredoxin reductases, play a central role in the functioning of the antioxidant system (Gu and Gao, 2022), counteracting the accumulation of hydroperoxides from cellular metabolism (Avery and Hoffman, 2018). In animals, the principal chemical form of Se is SeCys, which is incorporated specifically into the active sites of selenoenzymes (Mehdi and Dufrasne, 2016).

Farm animals are particularly subject to high levels of oxidative stress that can affect their performance and product quality (meat, eggs, and milk) (Edens and Sefton, 2016). In poultry, heat is an important stressor, increasingly experienced worldwide and not only in the hot regions of the world. Dietary Se can positively affect growth performance, antioxidation, and immune function in heat stressed broilers (Liao et al., 2012).

Protective antioxidant activity of selenoproteins is also important for animal reproduction, to prevent negative consequences of overproduction of free radicals in mammalian spermatozoa. Indeed, mammalian spermatozoa contain high proportions of polyunsaturated fatty acids, which are susceptible to free radical attack and lipid peroxidation. Impaired spermatogenesis due to Se deficiency has been reported in several animal species, including pigs (Marin-Guzman et al., 2000). Natural antioxidants (vitamin E, ascorbic acid), together with antioxidant enzymes (superoxide dismutase and glutathione peroxidase) are necessary to build an integrated antioxidant system in mammalian semen (Surai and Fisinin, 2015).

To date, no recommended daily amount of Se has been set in the European Union and the maximum authorised level is 0.5 mg/g for total Se. The National Research Council provides guidelines recommending a daily amount of Se of 100 μg/kg of DM for beef cattle and calves, and 300 μg/kg DM for dairy cows (NRC, 2016). For poultry, daily amounts of 150 to 200 μg/kg of DM (which in some cases can reach 300 μg/kg DM) are recommended. These levels, in the European Union, reach up to a maximum of 500 μg/kg DM (Mavromichalis, 2021). For swine, the daily recommendation is 150 μg/kg DM (sows and fattening pigs) and up to 300 μg/kg DM (weaning piglets) (NRC, 2012).

Selenium is essential not only for terrestrial animals but also for several farmed fish species, including salmonids, even if the biochemical functions of selenoproteins in fish are poorly understood (Lall and Kaushik, 2021). However, beneficial effects and toxicity of dietary Se supplementation in fish are well documented (Hosnedlova et al., 2017; Suttle, 2010). For example, the effects of dietary Se also show an increase in expression of selenoprotein P in rainbow trout and zebrafish (Fontagné-Dicharry et al., 2015; Pacitti et al., 2015; Penglase et al., 2015; Wang et al., 2018).

### 2.2 Selenium-containing feed additives: legal framework

Within the European Union, different Se-containing feed additives are used in animal nutrition. Prior to their use, these products have to undergo a pre-market authorisation as specified in Regulation (EC) No. 1831/2003 (European Union, 2003). This regulation classifies feed additives into various categories, which are further split into different functional groups. Se-containing feed additives belong to the category “nutritional additives” and the functional group “compounds of trace elements”, abbreviated as 3b. One of the key elements of this procedure is the separation of risk assessment conducted by the European Food Safety Authority (EFSA) and the risk management, which involves the final decision on approval or denial of the request for authorisation. The latter task falls under the responsibility of the European Commission, which issues Commission Implementing Regulations containing feed additive-specific details of the authorisation or the denial. The European Commission also has a European Union feed additive register (European Union, 2022a).

Applicants seeking authorisation need to send: (1) an application to the European Commission, (2) a dossier containing technical and scientific details on the product to EFSA, and (3) 3 samples of the product to the European Union Reference Laboratory for Feed Additives (EURL-FA). Legislation therefore requires that applicants have to present suitable analytical methods that are subsequently evaluated by the EURL-FA, supported by a network of member states' national reference laboratories. Details of the operation of the EURL-FA are given in a previous publication (von Holst et al., 2016). Once the EURL-FA has finalised the evaluation of the analytical methods, the corresponding report is published on the EU Science Hub (European Union, 2022b).

Table 1 shows all currently authorised feed additives containing Se as trace element, along with the corresponding Commission Implementing Regulations. Each regulation contains all relevant details individually for each authorised feed additive, i.e. (1) the reference to the product specific EFSA opinion, (2) the characterisation of the feed additive, (3) the conditions of use such as legal limits, and (4) a short description of the analytical methods. When authorising the product, the European Commission attributes an additive-specific identification number that needs to be used, e.g. for labelling, and helps the user identify the correct additive. The feed additive can be identical to the active substance such as with sodium selenite, or different as with Se-yeast. In the latter case, the active substance is SeMet, while the feed additive is the Se-yeast. The authorisation of Se-yeast is strain specific, thus requiring a company to submit a new application when the yeast strain concerned is not yet authorised.

**Table 1:** Feed additives containing selenium (Se) as an active substance and authorised in the European Union (up to 2022).

Feed additive	Identification number	Product specifications	Additive substance	Commission implementing regulation
Sodium selenite	3b801	Minimum Se content: 45%	Sodium selenite	(EU) 2019/49
Coated granulated sodium selenite	3b802	Preparation containing various materials; Range of Se content: 1% to 4.5%	Sodium selenite	(EU) 2019/49
Sodium selenate	3b803	Minimum Se content: 41%	Disodium selenate	(EU) 2020/37
Se-yeast *Saccharomyces cerevisiae* CNCM I-3060	3b810	Content of Se: 2,000 to 4,000 mg/kg; Organic Se > 97% to 99% of total Se; SeMet > 63% of total Se	SeMet produced by *Saccharomyces cerevisiae* CNCM I-3060	(EU) 2019/804
Se-yeast *Saccharomyces cerevisiae* NCYC R397	3b811	Content of Se: 2,000 to 3,500 mg/kg; Organic Se > 98% of total Se; SeMet> 63% of total Se	SeMet produced by *Saccharomyces cerevisiae* NCYC R397	(EU) 2019/804
SeMet produced by *Saccharomyces cerevisiae* CNCM I3399	3b812	Content of Se: 2,000 to 3,500 mg/kg; Organic Se > 97 to 99% of total Se; SeMet > 63% of total Se	SeMet produced by *Saccharomyces cerevisiae* CNCM I-3399	(EU) 2020/2117
SeMet produced by *Saccharomyces cerevisiae* NCYC R646	3b813	Range of Se content: 1,000 to 2,650 mg/kg; Organic Se > 98% of total Se; SeMet > 70% of total Se	SeMet produced by *Saccharomyces cerevisiae* NCYC R646	(EU) 2013/427
Hydroxy-analogue of SeMet	3b814	Solid and liquid preparation of Hydroxy-analogue of SeMet; Content of Se: 18,000 to 24,000 mg Se/kg	Organic Se from hydroxy-analogue of SeMet	(EU) 2013/445
L-SeMet	3b815	Solid preparation of L-SeMet with a Se content <40 g/kg	Organic Se in form of L-SeMet (2-amino-4-methylselanyl-butanoic acid)	(EU) 2014/121

Se-yeast = Se-enriched yeast; SeMet = selenomethionine.

All regulations authorising Se-containing feed additives establish a legal limit of 0.5 mg/kg for total Se in complete feed, thus requiring the feed operator to also take into account other sources of Se from the various feed materials in the final compound feed. When using Se in its organic form, an additional provision foresees that the maximum supplementation with organic Se is 0.2 mg/kg in complete feed.

The addition of essential nutrients to animal feeds is regulated in the USA by the Food and Drug Administration in the Federal Food, Drug and Cosmetic Act. These essential nutrients are thus regulated as food additives or through qualification for the generally recognized as safe list. In the USA, according to the Code of Federal Regulations, Part 573 (21 CFR, 573.920) – Food additives permitted in the feed and drinking water of animals, 4 different sources are recognized: (1) sodium selenite or sodium selenate, (2) controlled-release sodium selenite bolus, (3) Se-yeast, and (4) SeMet hydroxy analogue (21 CFR, 573.920). Selenium, provided as inorganic form or organic form (Se-yeast and SeMet hydroxy analogue) is authorised at a legal limit of 0.3 mg/kg in complete feed for chickens, turkeys, cattle (beef and dairy cattle), and swine. Moreover, sodium selenite/selenate is also recognised for sheep and ducks at a level not exceeding 0.3 mg/kg (21 CFR, 573.920).

Selenium, as SeMet hydroxy analogue, needs to meet additional specifications such as: arsenic, not more than 2 mg/kg, cadmium, lead, and mercury not more than 1 mg/kg (21 CFR, 573.920).

On the other hand, controlled-release sodium selenite bolus is for use only in beef and dairy cattle that are over 3 mo of age or over 91 kg in body weight in order to ensure animal health. Specifically, only one bolus containing 360 mg of Se (as sodium selenite) is administered orally to each animal in 120 d (21 CFR, 573.920).

Similarly to the USA, Canada also applies a legal limit of 0.3 mg/kg Se added to compound feed for swine, chickens, turkeys, ducks, geese, beef cattle, growing dairy cattle (non-lactating heifers, bulls, and steers), sheep and goats, as well as in the dry material of milk replacers for calves, lambs, colts and piglets (Government of Canada, 2022). On the other hand, in complete feeds for rabbits and salmonid fish, the legal level should not exceed 0.1 mg/kg Se (Government of Canada, 2022).

However, upon combining different regulatory documents available in the USA, Canada and in the European Union irrespective of the different sources, supplementation values seem to be very close.

## 3 Selenium deficiency

The benefits of Se for humans and other mammals were identified for the first time in the 1950s by Klaus Schwartz and Calvin Foltz, who demonstrated that dietary Se protected rats against liver necrosis (Schwarz and Foltz, 1957). Since then, 25 selenoproteins have been identified, half of which have been characterised (Rayman, 2002) and the role of Se as a trace mineral nutrient in human and animal health has become better understood.

Many pathological conditions arise when Se levels drop under recommended dose for a long period, and one of these is Keshan disease. This disorder arises when dietary Se uptake is not met and occurs predominately in children and women of child-bearing age (Koller and Exon, 1986). Selenium deficiency does not depend only on eating habits and often finds a reason due to geographical location. Since the Se content of food is highly dependent on the amount of Se in the soil, Se deficiency is a public health issue that varies from country to country but also from region to region (Avery and Hoffman, 2018). In fact, there are countries where Se deficiency in the population is sporadic, such as the USA and Canada (Avery and Hoffman, 2018). On the other hand, people living in China, New Zealand, and parts of Europe and Russia consistently present with insufficient Se intake due to low levels of Se in the soil and, for this reason, in food (Ye et al., 2020). For example, several authors report that most soils in China are characterised with Se deficiency, although there are some areas such as Enshi in Hubei with a concentration above the global average (Huang et al., 2021).

Selenium is an essential trace element that is also indispensable for the wellbeing of livestock. Selenium deficiency in livestock provokes different types of diseases with potential enormous economic losses to producers each year. These diseases range from "white muscle disease" to numerous lesser known conditions, often referred to as Se-responsive diseases resulting in reduced weight gain, diarrhoea, abortions, and diminished fertility (Koller and Exon, 1986). Clinical manifestations of Se deficiency have been found in both monogastric and polygastric animals, with different types of problems which vary depending on the animal species involved. In chickens, Se deficiency can cause several diseases as pancreas atrophy, diarrhoea, reproductive dysfunction, and immune or nerve damage (Yang et al., 2016). A Se-deficient diet causes changes in immune function and may cause oxidative stress in the thymus of chickens with inhibition of bursal and thymic growth (Khoso et al., 2017). In cows, there may be problems with placental retention (Underwood and Suttle, 1999), while in bulls the viability of the sperm is negatively affected, in particular it is reduced (Slaweta et al., 1988). Reproductive problems have also been found, for example, in boars where there is impaired development of spermatozoa (Marin-Guzman et al., 1997). In sows, the lack of this mineral leads to reduced litter size and, also in piglets and in hens, the conception rate and egg production is affected (Underwood and Suttle, 1999). Finally, high embryonic mortality was found in sheep (Hartley, 1963; Wilkins and Kilgour, 1982) and wool production was negatively affected (Gabbedy, 1971).

Since humans and animals rely on plants as the main source of Se in the diet, in regions where the soil is deficient in this element, different strategies are applied in order to supply the population with sufficient Se, such as the use of Se fertilizers or Se supplementation of feed ingredients for farm animals (Dumont et al., 2006).

## 4 Biofortification

The purpose of biofortification is to increase essential micronutrients and other health-promoting compounds in the edible parts of plants or animals with the aim of improving the nutritional quality of diets (Schiavon et al., 2020). This increase can be achieved by mineral fertilizer or feed supplementation (D'Amato et al., 2020).

Since Se is deficient in different regions of the planet, Se biofortification of feed/food is indispensable. Despite the fact that exposure of Se to an adult human via the diet varies between 11 and 5,000 μg Se/d over the world, the average dietary intake usually falls within the range of 20 to 300 μg Se/d (Patrick, 2004). International agencies have set the recommended Se dietary intake of 30 to 55 μg Se/d as the safe level to avoid Se deficiency. However, according to Zhou et al. (2020), more than 15% of the world's population already suffers from Se deficiency, leading to serious medical complications including cataracts, endothelial dysfunction, cardiovascular disease, cardiomyopathy, poor immune function, and even cancer.

The production of Se-enriched feed/food is still challenging but is necessary to exploit the nutraceutical potential of these products in order to address this long-standing public health problem.

### 4.1 Selenium fertilizers

The easiest way to enrich products of plant origin is to use Se fertilizers. Although several studies have been conducted worldwide (Alfthan et al., 2015; Grant et al., 2007; McLaren and Clucas, 2006; Yan et al., 2021; Yuan et al., 2022), this strategy is mainly used in Finland, where the problem of Se deficiency in soil is widespread. Biofortification can be carried out in different ways, such as application on leaves (Graham, 2018; Kápolna et al., 2007; Ros et al., 2016; Xiong et al., 2018) or directly in the ground (Broadley et al., 2010; Larsen et al., 2006; Ros et al., 2016).

The contribution of Se to the plant, however, depends on the ability of the plant itself to accumulate Se, and on the local environment (Hossain et al., 2021). This means that the type of soil and the ability of the plant to absorb/accumulate Se place a limit on this practice. In fact, the addition of Se fertilizers to the soil is an appropriate way to biofortify foodstuffs. However, high amounts of Se need to be applied to the soil to obtain Se concentrations in plants equal to other fertilization methods (Broadley et al., 2010; Puccinelli et al., 2017).

The direct addition of Se fertilizers to soils thus focuses concern on the high Se levels in the environment, which act as an environmental contaminant (Lichtfouse et al., 2022). The application of Se fertilizer to the soil is connected with several other problems, such as adsorption with soil colloidal surfaces, resulting in less Se available to the plants. Other techniques, such as the application on leaves, depend on the characteristics of the plant or Se fertilizers used (Hossain et al., 2021). For instance, results of spraying on leaves depend on the characteristics of the leaf and fruit surface, such as the presence of hairs, the characteristics of the epicarp, and the chemical composition of the epicuticular wax (Puccinelli et al., 2017). Consequently, the tendency to biofortify feed/food ingredients has become increasingly widespread.

### 4.2 Organic vs inorganic selenium

Organic forms of Se, for example Se amino acids such as SeMet, are more bioavailable than inorganic forms, such as selenite and selenate (Fontagné-Dicharry et al., 2015; Guyot et al., 2007; Lorentzen et al., 1994; Marković et al., 2018; Paiva et al., 2019; Słovińska et al., 2011; Tian et al., 2006; Wang and Xu, 2008; Zhang et al., 2020). Organic forms are also better absorbed because they are absorbed into the intestinal tract through a mechanism of transport of amino acids, unlike inorganic Se, which is absorbed by a simple diffusion process (Gu and Gao, 2022).

However, the absorption efficiency of Se depends not only on the form in which Se is supplied but also on animal species involved and differs between ruminant and non-ruminant animals (Schlegel et al., 2008). For instance, absorption of inorganic Se provided as sodium selenite orally administered as [^75^Se]selenite was absorbed less efficiently than the labelled [^75^Se]SeMet as indicated by the higher percentage of Se isotopes in the gastrointestinal tract of chicks, measured at 43.1% for [^75^Se]selenite and 60.9% for [^75^Se]SeMet (Humaloja and Mykkänen, 1986). On the other hand, for ruminants the absorption rate of inorganic Se provided as sodium selenite in the small intestine is very low, with values around 30% or less. In fact, several authors have reported poor utilization/absorption of inorganic dietary Se in ruminants; absorption of inorganic ^75^Se in steers was estimated to be only 13% (Costa et al., 1985) and a similar true Se absorption was reported in non-lactating cows (from 10% to 16%) fed hay supplemented with inorganic Se (Koenig et al., 1991; Koenig and Beauchemin, 2009). This may be related to the reducing characteristics of the rumen or the activity of rumen microorganisms that reduce sodium selenite to insoluble elemental Se (Galbraith et al., 2016).

Absorption is not the only parameter to take into account when comparing organic and inorganic Se forms. Selenium supplied in inorganic form has high toxicity, low transfer from the animal to the resulting products (milk, eggs, and meat), and is not stored in the body (Dalgaard et al., 2018; Edens and Sefton, 2016). Inorganic species are more toxic for the organism than Se amino acids (Arshad et al., 2021; Khalili et al., 2020; Kim and Mahan, 2001; Schrauzer, 2000). Supplementation with selenite or selenate could increase Se levels in the tissues to toxic levels, with consequent selenosis. Inorganic Se is not only poorly absorbed but also poorly transferred to animal products. For example, in poultry sodium selenite is poorly transferred to the egg and developing embryo (Surai, 2006), and thus has a limited ability to improve antioxidant defence against hatching-related oxidative stress (Surai et al., 2016).

Given the better absorption and lower toxicity, the biofortification of feed with Se in organic form is preferred although selenite/selenate supplementation remains very widespread, as sodium selenite/selenate is cheaper than Se-yeast.

The following subsections detail the different forms of Se supplementation, both those commercially available such as Se-yeast, and the possible new frontiers in supplementation such as Se-enriched insects and Se nanoparticles (SeNP).

#### 4.2.1 Se-enriched yeast

Se-yeast is a dried and non-viable yeast, mainly derived from *Saccharomyces cerevisiae*, grown in a medium made up of principally cane molasses and inorganic salts of Se. When the yeast is grown in a Se-enriched medium, it absorbs Se and, during fermentation, converts Se into several organic compounds, of which SeMet is typically the main one (Cattaneo et al., 2008).

Although many of the works in the literature report the ability of yeasts to accumulate Se up to 3,000 mg/kg (Kieliszek and Błażejak, 2013; Lynch et al., 2018), the formation of different Se species during yeast metabolism must be taken into account (Álvarez-Fernández García et al., 2020). In this regard, Se-yeast are an important source of organic Se for livestock. Consequently, Se-yeast quality is typically based on the assessment of the percentage SeMet (usually >60%) that usually is complementary to the absence (<2%) of both selenite and selenate (Bodnar et al., 2016; Schrauzer, 2006).

Nowadays, the total determination of Se in Se-yeast is no longer a problem as it can be routinely measured using inductively coupled plasma-mass spectrometry (ICP-MS) or inductively coupled plasma optical emission spectroscopy (ICP-OES) after mineral digestion of the sample (Álvarez-Fernández García et al., 2020; Amoako et al., 2007; Goenaga Infante et al., 2004; Kotrebai et al., 2000). Moreover, results can be validated by analysing some of the available reference materials such as SELM-1, produced by the Institute for National Measurement Standards, National Research Council of Canada (Álvarez-Fernández García et al., 2020). On the other hand, the characterization of other Se-metabolic products, even at trace levels, represents a hot topic since the biological actions of Se depend not only on the amount but also on the form of the Se species (Álvarez-Fernández García et al., 2020). Difficulties related to analytical methods make the identification and characterisation of all the Se species involved a real challenge. Suitable analytical methods for determining Se species are thus needed and are increasingly used.

In this context, speciation analyses are becoming increasingly popular for nutritionally important minerals such as Se. Analytical methods based on high performance liquid chromatography and inductively coupled plasma-mass spectrometry (HPLC-ICP-MS) and high-performance liquid chromatography-electrospray ionization tandem mass spectrometry (HPLC-ESI-MS/MS) are commonly used in this field (Álvarez-Fernández García et al., 2020; Goenaga Infante et al., 2004; Kotrebai et al., 2000). However, the variability in terms of Se content and Se species among commercially available Se-yeast, as well as the lack of knowledge regarding the identity of all the Se species, means that characterizing Se compounds in Se-yeast is difficult.

The amount of organic Se, especially SeMet, has been investigated in some studies (Bierla et al., 2012; Mester et al., 2006). The currently available analytical methods based on HPLC-ICP-MS allow the determination of SeMet with relatively high confidence due to the availability of a certified reference material, SELM-1 (Bierla et al., 2012; Hinojosa Reyes et al., 2006; Mester et al., 2006). For instance, Bodnar et al. (2016) found values between 60% and 70% for SeMet. However, other authors have reported a great variability in SeMet content in several Se-yeast products, with values ranging from 54% to 60% up to 85% (Ip et al., 2000; Rayman, 2004; Schrauzer, 2006). These results highlight how the great variability between Se-yeast production technologies represents an additional source of variation in the composition of Se-yeast supplements found on the market.

Regarding the content in inorganic Se, the HPLC-ICP-MS technique is one of the most used for the determination of the residual selenite and selenate in Se-yeast. Both these forms should represent less than 2% of total Se in yeast, indicating a proof of an “organic” character of Se-yeast (Jiménez-Lamana et al., 2018). To date, despite the advances in analytical methodologies, the Se mass balance of all the species identified rarely exceeds 90% of the total Se in commercial Se-yeast products (Jiménez-Lamana et al., 2018), and thus, the remaining 10% are still a great challenge. One of the compounds not yet characterized is elemental Se (Se(0)). Several authors have reported in the literature the synthesis of SeNP by microorganisms, such as bacteria or fungi, due to reduction of selenite to Se(0) (Kessi et al., 1999; Tugarova et al., 2014; Wadhwani et al., 2016), although only few papers have described the formation of Se(0) by yeast cell metabolism (Bierla et al., 2012; Kieliszek et al., 2015; Tarze et al., 2007). To the best of our knowledge, nano-sized deposits due to Se(0) have been identified in yeast cells only in recent years and only in a few studies (Álvarez-Fernández García et al., 2020; Jiménez-Lamana et al., 2018) without being quantified. The determination of Se(0) remains challenging due to its low solubility in water and, therefore, it is difficult to extract with the common protocols used for speciation analysis. This issue can be overcome, as reported by Vacchina et al. (2021), by converting Se(0) into a soluble compound, selenosulfate, after reaction with an excess of sodium sulfite at high temperature.

**Fig. 3:**
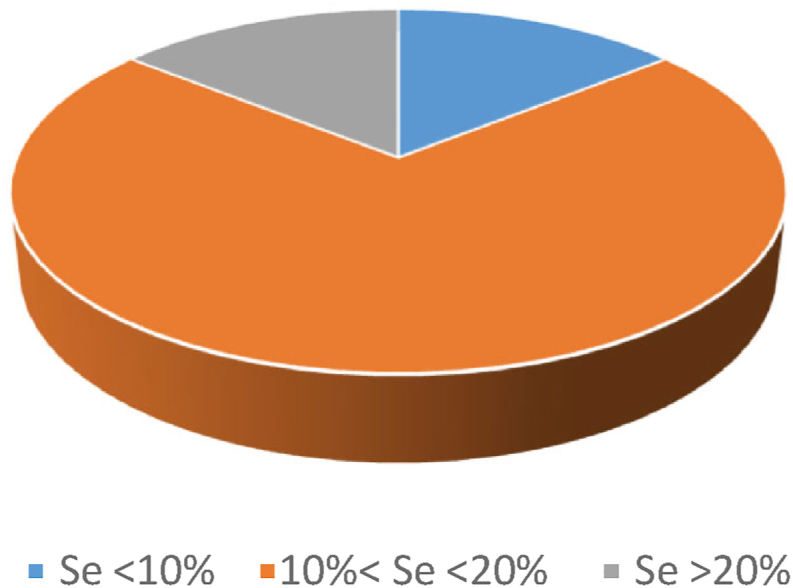
Selenium-enriched yeast grouped per elemental Se (Se(0)) percentage. One sample is below 10% Se(0) compared to total Se, 5 samples between 10% and 20% Se(0), and 1 sample above 20% Se(0) (adapted from Vacchina et al., 2021).

In fact, Vacchina et al. (2021) applied their new method to a set of 7 Se-yeast samples for the determination of Se(0). Elemental Se was recovered in all 7 different batches of commercial yeasts analysed (Vacchina et al., 2021). Most of the yeasts analysed in their study showed between 10% and 20% of Se(0) (Fig. 3). These values are considered very high compared with typical values of inorganic Se, i.e. around 2% to 3% (Bierla et al., 2012; Schrauzer, 2006). These results must be considered with caution; in fact, they derive from a single study conducted on Se-yeast in which Se(0) was analysed (Vacchina et al., 2021). Moreover, the latter has been carried out on only a small number of samples compared to the different Se-yeast supplements available on the market (Vacchina et al., 2021). Thus, further studies, possibly based on validated methods and carried out on a wider range of samples, are required to better understand the organic species present, even at trace levels, as well as to better evaluate the composition of inorganic Se in these products.

#### 4.2.2 Se-enriched insects

Several studies have highlighted how insects can be seen as an alternative protein source for farm animals (Pinotti et al., 2019; Spranghers et al., 2017). The most widespread species in this sense are *Hermetia illucens* (also called black soldier fly [BSF]), *Musca domestica* (common housefly) and *Tenebrio molitor* (yellow mealworm) (Pinotti et al., 2019; Pinotti and Ottoboni, 2021). Among these, the BSF species is certainly the most studied. Purchke et al. (2017) found that BSF show high bioaccumulation factors for certain heavy metals such as lead (Pb) and cadmium (Cd). In particular, the study highlights bioaccumulation factors of 2.3 and 9 for Pb and Cd, respectively (Purchke et al., 2017). Insects are therefore naturally able to accumulate metals and this characteristic could be used for the production of Se-enriched insects. Biofortification of Se in insects would be valuable in terms of exploring alternatives, for example, to the addition of Se in feed for the creation of premium speciality feed.

Despite several studies investigating the accumulation of heavy metals or other nutrients (e.g. unsaturated fatty acids) by insects, to the best of our knowledge Ferrari et al. (2022) is the only article in the literature to study the accumulation of Se by BSF prepupae, also evaluating Se species and not only the accumulation of total Se.

In the above study, Ferrari et al. (2022) observed that when BSF larvae were fed with a diet enriched with selenite, the amount of Se in the prepupae resulted in a consistent increase, despite the lack of bioaccumulation. Adding sodium selenite (with a 22% increase in Se in the diet) resulted in a total Se concentration in prepupae that was more than 3 times higher than the concentration of Se in prepupae fed the control diet (see Table 2). Although not exactly Se bioaccumulation, Ferrari's study emphasizes how, starting from a similar diet, adding a source of inorganic Se leads to a significant increase in Se, and in particular organic Se (Ferrari et al., 2022).

**Table 2:** Analysed selenium (Se) concentrations in test substrates and in corresponding black soldier fly (BSF) prepupae^1^ and calculated bioaccumulatioin factor (BAF), and Se increase ratio relative to control group prepupae (adapted from Ferrari et al., 2022).

Rearing substrate	Se in the substrate, mg/kg	Se in the prepupae, mg/kg	BAF^2^	Se increase expressed as ratio relative to control group prepupae
Control diet	1.33	0.41	0.31	–
Algae (AN30) diet	1.01	0.21	0.21	−50%
Se diet	1.63	1.17	0.72	+285%

1 Expressed as milligrams of Se per kilogram DM substrate or prepupae.

2 Calculated as in Purchke et al. (2017).

Black soldier fly larvae are considered a potential good quality feed ingredient. In fact, BSF larvae have a crude protein content (38.0% to 60.4%) comparable to fishmeal (60.5% to 65%) or soybean (42.0% to 47%), making them a valuable alternative protein source (Arango Gutiérrez et al., 2004) already used in several studies in the diet of various animal species (Affedzie-obresi et al., 2020; Bruni et al., 2020; Star et al., 2020; Tan et al., 2020). Black soldier fly larvae could thus be enriched with Se to create a premium speciality feed that could complement Se-yeast. In fact, the Se content in BSF larvae is lower than the Se content in Se-yeast, however, insects could be an important source of Se and at the same time an alternative protein source, due to the higher biomass compared to yeast.

**Fig. 4:**
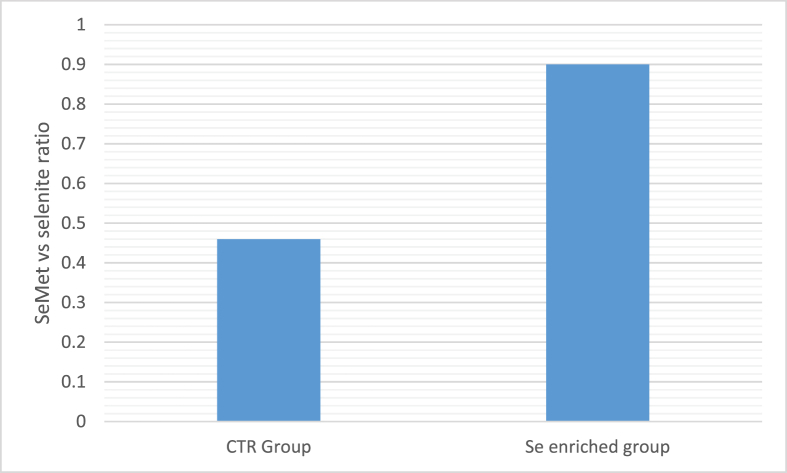
The ratio of selenomethionine (SeMet) to selenite in black soldier fly (BSF) prepupae fed the control diet (CTR group) vs Se-enriched diet (Se enriched group).

Selenium speciation analysis thus represents a powerful tool not only in Se-yeast but also in insects to evaluate the Se species present. Since different Se forms have different levels of bioavailability as well as different metabolic pathways (D'Amato et al., 2020), the determination of organic versus inorganic Se is thus important, as well as the determination of the different organic Se species. In prepupae fed a Se-enriched diet, around 50% of the Se was in the form of inorganic selenite and selenate, while 0.55 mg/kg was in the form of SeMet (Ferrari et al., 2022). Comparing the prepupae fed the Se-enriched diet and the control diet, the addition of sodium selenite led to an increase in both inorganic Se and SeMet in prepupae. However, the increase in SeMet was much more marked than the increase in inorganic Se (Fig. 4).

How Se tends to be distributed in insects is of great importance since chitin is generally removed in the production of insect-based feed. In a previous study by our research group, a sequential extraction was applied, digesting the sample first with protease prior to chitinase in order to digest the non-soluble residue and to obtain complete Se extraction (Ferrari et al., 2022). The Se content in the extract with protease then reveals how much Se is accumulated in the internal tissues and, therefore, is potentially available for the creation of premium speciality feed. The results show that in the prepupae fed Se-enriched diet, almost 40% of the total Se is contained in the extract with protease. In contrast, the prepupae fed the control diet show approximately 47% of their total Se content in the extract (Ferrari et al., 2022). For both diets, SeMet represents a huge amount of organic Se, with several other compounds in a very limited amount. Based on these results it can be speculated that 1 kg of insect material might contribute significantly in covering regular farm animal dietary requirements of Se (about 0.1 to 0.3 mg/kg in several animal species) (Ferrari et al., 2022).

The presence of both organic and inorganic Se could be a valuable characteristic for BSF. In fact, organic Se is better absorbed and also less toxic (Whanger, 2002). However, dietary SeMet is partially incorporated non-specifically into muscle protein in place of methionine (Wastney et al., 2011), acting as a source of stored Se in the muscle that is mobilised when Se is needed to provide SeCys (Edens and Sefton, 2016; Ip and Hayes, 1989). On the other hand, selenite and selenate are readily absorbed and involved in the synthesis of SeCys, and thus incorporated into selenoproteins which play an important role in the antioxidant defence system of the organism (Ip and Hayes, 1989; Shimizu et al., 2021). However, a better understanding of the organic species involved could help evaluate the effects when BSF is used as a Se supplement, as well as studies on increasing Se uptake by insects. These scenarios represent a first step in the evaluation of Se-enriched insect feed which need to be proven in a feeding trial in targeted species.

#### 4.2.3 Selenium nanoparticles

Nanotechnology is an innovative solution to provide nutrients for animals and to protect health, thus improving animal production systems (Horky et al., 2016) as well as the quality of products of animal origin. Nanoparticles have unique physicochemical properties such as small dimensions (1 to 100 nm), high stability, hydrophobicity, and large surface area (Gu and Gao, 2022; Malyugina et al., 2021). For instance, the hydrophobicity of nanoparticles ensures good dispersion in water or serum as well as enhanced interaction with cell membranes (Fratoddi, 2017). On the other hand, the size of nanoparticles facilitate passage through the stomach wall and diffusion into the body's cells which occurs faster than common elements with larger particle sizes.

Several studies have investigated using SeNP as a new source of Se. Selenium administered in the form of nanoparticles, supplemented at 0.3, 3, and 6 mg Se/kg DM, improved the content of Se in the blood and tissues in sheep, leading also to better rumen fermentation and better feed use (Shi et al., 2011). Similarly, 4 mg Se/kg DM of SeNP, showed an improvement in rumen fermentation and feed conversion efficiency in sheep compared to Se-yeast (Xun et al., 2012). Several studies on broilers have reported an improvement in growth (Ahmadi et al., 2018; Senthil Kumaran et al., 2015), intestinal health (Gangadoo et al., 2018) and antioxidant capacity (Hassanin et al., 2013; Senthil Kumaran et al., 2015) when SeNP supplementation was used in the diet. Fuxiang et al. (2008) found that antioxidant status and immunity were higher in birds fed diets containing 0.15 to 1.2 mg/kg SeNP. Similarly, dietary supplementation of 0.25 mg/kg SeNP for laying hens improved the GSH-Px activity (Radwan et al., 2015), thus upregulating the antioxidant status of birds exposed to heat stress compared to birds fed diets enriched with other forms of Se (Senthil Kumaran et al., 2015).

Compared to selenite and selenate, SeNP are more biocompatible and less toxic to animal organisms (Skalickova et al., 2017). In fact, diets containing SeNP at doses from 0.2 to 0.5 mg/kg showed a significant improvement in carcass characteristics and growth, without adverse effects on internal organs (Ahmadi et al., 2018). In addition, Hu et al. (2012) reported that broilers fed diets containing 0.15 to 1.20 mg/kg SeNP showed a significant improvement in body weight gain compared to broilers fed diets containing 0.30 mg/kg inorganic Se.

Improvements due to the effects of SeNP have been reported in several fish species. Growth performance (Jahanbakhshi et al., 2021), intestinal health (Dawood, 2021; Ghazi et al., 2021), and antioxidant status (Longbaf Dezfouli et al., 2019), and immune response (Kohshahi et al., 2019) of aquatic animals supplemented with SeNP were improved. For instance, SeNP supplementation (0.4 to 0.8 mg Se) improved intestinal health, feed use, and growth performance in Nile tilapia (Ghazi et al., 2021). Similarly, Ibrahim et al. (2021) reported an enhancement in the growth performance and feed efficiency after SeNP supplementation (0.4 to 0.8 mg Se) in Nile tilapia.

However, excessive doses or prolonged supplementation with SeNP can have negative effects in animal organisms and can be toxic. Nanoparticles’ toxicity varies widely between different species (Wadhwani et al., 2016). For instance, the supplemental level of SeNP in broiler diets should not exceed 1.0 mg/kg, and optimal supplementation levels range from 0.3 to 0.5 mg/kg (Cai et al., 2012). Wang and Xu (2008) investigated the toxicity of SeNP in broilers fed higher levels (4.25 mg/kg), concluding that high levels induced toxicity due to cellular stress, altering carbohydrate and fatty acid metabolism. Gangadoo et al. (2018) found that an intermediate concentration of SeNP (0.9 mg/kg) performed better than lower (0.3 mg/kg) and higher (1.5 mg/kg) levels, improving the gut health by increasing the abundance of beneficial bacteria, such as *Lactobacillus* and *Faecalibacterium*, and short-chain fatty acid production, in particular butyric acid.

Based on the studies reported above, compared to sources of inorganic Se, the integration of Se through the use of nanoparticles has numerous benefits when introduced into the diets of animals. However, high doses of SeNP lead to the hyperaccumulation of Se in the tissues causing oxidative stress or toxicity (Gu and Gao, 2022). Therefore to increase the benefits for livestock, SeNP should be included in such a way that the feed is as balanced as possible.

Further studies are required to better understand the potential toxicity of SeNP and to provide insights into the mechanism of action in several animal species. The appropriate levels of SeNP in diets as well as the composition, particle size, and synthesis strategies need to be evaluated for a better understanding of this technology.

## 5 Conclusions and perspectives

Selenium deficiency, which causes many diseases for both humans and animals, is a global issue. In fact, the Se content of food is highly dependent on the amount of Se in the soil, which varies from country to country but also from region to region. Selenium biofortification, which can prevent various disorders, offers an effective approach, however, it still requires further research.

The accumulation of Se in plant products is due to the ability of plants to transform inorganic forms into organic forms. In this sense, feed/food biofortification could be an excellent way to combat Se deficiency rather than using Se fertilizers.

Se-yeast provide economic and environmental advantages over traditional inorganic Se. Lower toxicity, combined with greater bioavailability, means that reduced amounts of Se can be used to supplement the Se-deficient diets of farm animals. However, new and interesting findings demonstrate that Se-yeast may contain less than 97% of total Se (and so less than 63% SeMet) due to the presence of elemental Se. To better characterise the proportion of inorganic and organic Se in yeasts, further studies should thus be performed, possibly on a wider range of samples.

Insects are a potential source of Se supplementation, as they combine the positive characteristics of Se in an organic form (better absorption and lower toxicity) with the ability of inorganic forms to take part in the synthesis of SeCys, which plays an important role in the antioxidant defence system of the organism. Insects could also be used as an alternative protein source in animal nutrition, thus reducing the use of traditional protein sources. The combined use of insects with Se-yeast could therefore be an excellent way of supplying Se. However, a better understanding of the organic Se species involved represents a first step in the evaluation of the efficacy of Se-enriched insects.

Selenium nanoparticles are a new type of supplementation with numerous benefits for the health of the animal and, at the same time, represent a sustainable alternative. Although there seem to be several beneficial effects for different species (ruminants, poultry, and pigs), the toxicity of Se must be taken into account.

In conclusion, insects and Se nanoparticles are relatively unknown sources of supplementation compared to Se-yeast and further research is thus necessary for a better understanding of these sources and to further optimize this technology.

## Author contributions

**Luciano Pinotti**, **Christoph von Holst**: Conceptualization. **Luca Ferrari**, **Rossella Abbate**, **Michele Manoni**, **Matteo Ottoboni**, **Alice Luciano**: Data curation, Writing – Original draft preparation, Visualization, Investigation. **Donata M.I.R. Cattaneo**: Supervision. **Luciano Pinotti**, **Donata M.I.R. Cattaneo**: Writing – Reviewing and Editing.

## Declaration of competing interest

We declare that we have no financial and personal relationships with other people or organizations that can inappropriately influence our work, and there is no professional or other personal interest of any nature or kind in any product, service and/or company that could be construed as influencing the content of this paper.

## References

[bib1] Adadi P., Barakova N.V., Muravyov K.Y., Krivoshapkina E.F. (2019). Designing selenium functional foods and beverages: a review. Food Res Int.

[bib2] Affedzie-obresi S., Adu-Aboagye G., Nkegbe E., Asuming-Bediako N., Ansah K., Mensah-Bonsu A., Sarpong D., Amegashie D., Kwadzo G.-M., Wallace P. (2020). Black soldier fly (*Hermitia illucens*) larvae meal as alternative protein in broiler production in Ghana. Ghana J Agric Sci.

[bib3] Ahmadi M., Ahmadian A., Seidavi A.R. (2018). Effect of different levels of nano-selenium on performance, blood parameters, immunity and carcass characteristics of broiler chickens. Poult Sci.

[bib4] Alfthan G., Eurola M., Ekholm P., Venäläinen E., Root T., Korkalainen K., Hartikainen H., Salminen P., Hietaniemi V., Aspila P., Aro A. (2015). Effects of nationwide addition of selenium to fertilizers on foods, and animal and human health in Finland: from deficiency to optimal selenium status of the population. J Trace Elem Med Biol.

[bib5] Álvarez-Fernández García R., Corte-Rodríguez M., Macke M., LeBlanc K.L., Mester Z., Montes-Bayón M., Bettmer J. (2020). Addressing the presence of biogenic selenium nanoparticles in yeast cells: analytical strategies based on ICP-TQ-MS. Analyst.

[bib6] Amoako P.O., Kahakachchi C.L., Dodova F.N., Uden P.C., Tyson J.F. (2007). Speciation, quantification and stability of selenomethionine, S-(methylseleno)cysteine and selenomethionine Se-oxide in yeast-based nutritional supplements. J Anal At Spectrom.

[bib7] Arango Gutiérrez G.P., Vergara Ruiz R.A., Mejía Vélez H. (2004). Compositional, microbiological and protein digestibility analysis of the larva meal of *Hermetia illuscens* L.(Diptera: Stratiomyidae) at Angelópolis-Antioquia, Colombia. Rev Fac Nac Agron.

[bib8] Arshad M., Ebeid H.M., Hassan F. (2021). Revisiting the effects of different dietary sources of selenium on the health and performance of dairy animals: a review. Biol Trace Elem Res.

[bib9] Avery J.C., Hoffman P.R. (2018). Selenium, selenoproteins, and immunity. Nutrients.

[bib10] Baldi A., Savoini G., Pinotti L., Monfardini E., Cheli F., Dell'Orto V. (2000). Effects of vitamin E and different energy sources on vitamin E status, milk quality and reproduction in transition cows. J. Vet. Med. A.

[bib11] Bierla K., Szpunar J., Yiannikouris A., Lobinski R. (2012). Comprehensive speciation of selenium in selenium-rich yeast. Trends Analyt Chem.

[bib12] Bodnar M., Szczyglowska M., Konieczka P., Namiesnik J. (2016). Methods of selenium supplementation: bioavailability and determination of selenium compounds. Crit Rev Food Sci Nutr.

[bib13] Broadley M.R., White P.J., Bryson R.J., Meacham M.C., Bowen H.C., Johnson S.E., Hawkesford M.J., McGrath S.P., Zhao F., Breward N., Harriman M., Tucker M. (2006). Biofortification of UK food crops with selenium. Proc Nutr Soc.

[bib14] Broadley M.R., Alcock J., Alford J., Cartwright P., Foot I., Fairweather–Tait S.J., Hart D.J., Hurst R., Knott P., Mcgrath S.P. (2010). Selenium biofortification of high-yielding winter wheat (*Triticum aestivum* L.) by liquid or granular Se fertilisation. Plant Soil.

[bib15] Bruni L., Randazzo B., Cardinaletti G., Zarantoniello M., Mina F., Secci G., Tulli F., Olivotto I., Parisi G. (2020). Dietary inclusion of full-fat *Hermetia illucens* prepupae meal in practical diets for rainbow trout (*Oncorhynchus mykiss*): lipid metabolism and fillet quality investigations. Aquaculture.

[bib16] Cai S.J., Wu C.X., Gong L.M., Song T., Wu H., Zhang L.Y. (2012). Effects of nano-selenium on performance, meat quality, immune function, oxidation resistance, and tissue selenium content in broilers. Poult Sci.

[bib17] Cattaneo D., Invernizzi G., Ferroni M., Agazzi A., Rebucci R., Baldi A., Dell'Orto V., Savoini G., Faye Bernard, Sinyavskiy Yuriy (2008). Impact of pollution on animal products.

[bib18] Code of Federal Regulations (CFR). https://www.ecfr.gov/current/title-21/chapter-I/subchapter-E/part-573/subpart-B/section-573.920 [accessed November 2022].

[bib19] Constantinescu-Aruxandei D., Frîncu R.M., Capra L., Oancea L. (2018). Selenium analysis and speciation in dietary supplements based on next-generation selenium ingredients. Nutrients.

[bib20] Costa N.D., Glled P.T., Sansom B.F., Symonds H., Allen W.M., Mills (1985). Trace elements in man and animals.

[bib21] Dalgaard T.S., Briens M., Engberg R.M., Lauridsen C. (2018). The influence of selenium and selenoproteins on immune responses of poultry and pigs. Anim Feed Sci Technol.

[bib22] D'Amato R., Regni L., Falcinelli B., Mattioli S., Benincasa P., Dal Bosco A., Pacheco P., Proietti P., Troni E., Santi C., Businelli D. (2020). Current knowledge on selenium biofortification to improve the nutraceutical profile of food: a comprehensive review. J Agric Food Chem.

[bib23] Dawood M.A. (2021). Nutritional immunity of fish intestines: important insights for sustainable aquaculture. Rev Aquac.

[bib24] Dumont E., Vanhaecke F., Cornelis R. (2006). Selenium speciation from food source to metabolites: a critical review. Anal Bioanal Chem.

[bib25] Edens F.W., Sefton A.E. (2016). Organic selenium in animal nutrition—utilisation, metabolism, storage and comparison with other selenium sources. J Appl Anim Nutr.

[bib26] European Union (2003). Regulation (EC) No 1831/2003 of the European Parliament and the council of 22 September 2003 on additives for use in animal nutrition. Off J Eur Union.

[bib27] European Union. https://food.ec.europa.eu/system/files/2022-08/animal-feed_additives_eu-register_1831-03_0.pdf, 2022a.

[bib28] European Union. https://joint-research-centre.ec.europa.eu/eurl-fa-eurl-feed-additives_en, 2022b.

[bib29] Ferrari L., Sele V., Silva M., Bonilauri P., De Filippo F., Selmin F., Ørnsrud R., Pinotti L., Ottoboni M. (2022). Biofortification of selenium in black soldier fly (*Hermetia illucens*) prepupae reared on seaweed or selenium enriched substrates. J Insects Food Feed.

[bib30] Fontagné-Dicharry S., Godin S., Liu H., Prabhu P.A.J., Bouyssiere B., Bueno M., Tacon P., Médale F., Kaushik S.J. (2015). Influence of the forms and levels of dietary selenium on antioxidant status and oxidative stress-related parameters in rainbow trout (*Oncorhynchus mykiss*) fry. Brit J Nutr.

[bib31] Fratoddi I. (2017). Hydrophobic and hydrophilic Au and Ag nanoparticles. Breakthroughs and perspectives. Nanomaterials.

[bib32] Fu L.H., Wang X.F., Eyal Y., She Y.M., Donald L.J., Standing K.G., Ben–Hayyim G. (2002). A selenoprotein in the plant kingdom: mass spectrometry confirms that an opal codon (UGA) encodes selenocysteine in Chlamydomonas reinhardtii glutathione peroxidase. J Biol Chem.

[bib33] Fuxiang W., Huiying R., Fenghua Z., Jinquan S., Jianyang J., Wenli L. (2008). Effects of nano-selenium on the immune functions and antioxidant abilities of broiler chickens. Chin Sci Bull.

[bib34] Gabbedy B.J. (1971). Effect of selenium on wool production, body weight and mortality of young sheep in Western Australia. Aust Vet J.

[bib35] Galbraith M.L., Vorachek W.R., Estill C.T., Whanger P.D., Bobe G., Davis T.Z., Hall J.A. (2016). Rumen microorganisms decrease bioavailability of inorganic selenium supplements. Biol Trace Elem Res.

[bib36] Gangadoo S., Dinev I., Chapman J., Hughes R.J., Van T.T.H., Moore R.J., Stanley D. (2018). Selenium nanoparticles in poultry feed modify gut microbiota and increase abundance of *Faecalibacterium prausnitzii*. Appl Microbiol.

[bib37] Ghazi S., Diab A.M., Khalafalla M.M., Mohamed R.A. (2021). Synergistic effects of selenium and zinc oxide nanoparticles on growth performance, hemato-biochemical profile, immune and oxidative stress responses, and intestinal morphometry of Nile Tilapia (*Oreochromis niloticus*). Biol Trace Elem Res.

[bib38] Goenaga Infante H., O'Connor G., Rayman M., Wahlen R., Entwisle J., Norris P., Hearn R., Catterick T. (2004). Selenium speciation analysis of selenium-enriched supplements by HPLC with ultrasonic nebulisation ICP-MS and electrospray MS/MS detection. J Anal At Spectrom.

[bib39] Government of Canada; https://inspection.canada.ca/animal-health/livestock-feeds/regulatory-guidance/rg-1/chapter-4/eng/1329341411340/1329341520337?chap=7 [accessed on November 2022].

[bib40] Graham L. (2018). Biofortification of cereals with foliar selenium and iodine could reduce hypothyroidism. Front Plant Sci.

[bib41] Grant C.A., Buckley W.T., Wu R. (2007). Effect of selenium fertilizer source and rate on grain yield and selenium and cadmium concentration of durum wheat. Can J Plant Sci.

[bib42] Gu X., Gao C. (2022). New horizons for selenium in animal nutrition and functional foods. Anim Nutr.

[bib43] Guyot H., Spring P., Andrien S., Rollin F. (2007). Comparative responses to sodium selenite and organic selenium supplements in belgian blue cows and calves. Livest Sci.

[bib44] Hartley W.J. (1963). Selenium and Ewe fertility. Proc N Z Soc Anim Prod.

[bib45] Hassanin K.M., Abd El-Kawi S.H., Hashem K.S. (2013). The prospective protective effect of selenium nanoparticles against chromium-induced oxidative and cellular damage in rat thyroid. Int J Nanomed.

[bib46] Hinojosa Reyes L., Marchante-Gayón J.M., García Alonso J.I., Sanz-Medel A. (2006). Application of isotope dilution analysis for the evaluation of extraction conditions in the determination of total selenium and selenomethionine in yeast-based nutritional supplements. J Agric Food Chem.

[bib47] Horky P., Ruttkay-Nedecky B., Nejdl L., Richtera L., Cernei N., Pohanka M., Kopel P., Skladanka J., Hloucalova P., Slama P., Nevrkla P., Mlejnkova V., Klusonova I., Kizek R., Adam V. (2016). Electrochemical methods for study of influence of selenium nanoparticles on antioxidant status of rats. Int J Electrochem Sci.

[bib48] Hosnedlova B., Kepinska M., Skalickova S., Fernandez C., Ruttkay–Nedecky B., Malevu T.D., Sochor J.S., Baron M., Melcova M., Zidkova J., Kizek R. (2017). A summary of new findings on the biological effects of selenium in selected animal species—a critical review. Int J Mol Sci.

[bib49] Hossain A., Skalicky M., Brestic M., Maitra S., Sarkar S., Ahmad Z., Vemuri H., Garai S., Mondal M., Bhatt R., Kumar P., Banerjee P., Saha S., Islam T., Laing A.M. (2021). Selenium biofortification: roles, mechanisms, responses and prospects. Molecules.

[bib50] Hu C., Li Y., Xiong L., Zhang H., Song J., Xia M. (2012). Comparative effects of nano elemental selenium and sodium selenite on selenium retention in broiler chickens. Anim Feed Sci Technol.

[bib51] Huang C., Wang H., Shi X., Wang Y., Li P., Yin H., Shao Y. (2021). Two new selenite reducing bacterial isolates from paddy soil and the potential Se biofortification of paddy rice. Ecotoxicology.

[bib52] Humaloja T., Mykkänen H.M. (1986). Intestinal absorption of 75Se-labeled sodium selenite and selenomethionine in chicks: effects of time, segment, selenium concentration and method of measurement. J Nutr.

[bib53] Ibrahim M.S., El-gendy G.M., Ahmed A.I., Elharoun E.R., Hassaan M.S. (2021). Nanoselenium versus bulk selenium as a dietary supplement: effects on growth, feed efficiency, intestinal histology, haemato-biochemical and oxidative stress biomarkers in Nile tilapia (*Oreochromis niloticus* Linnaeus, 1758) fingerlings. Aquac Res.

[bib54] Ip C., Hayes C. (1989). Tissue selenium levels in selenium-supplemented rats and their relevance in mammary cancer protection. Carcinogenesis.

[bib55] Ip C., Birringer M., Block E., Kotrebai M., Tyson J.F., Uden P.C., Lisk D.J. (2000). Chemical speciation influences comparative activity of selenium-enriched garlic and yeast in mammary cancer prevention. J Agric Food Chem.

[bib56] Jahanbakhshi A., Pourmozaffar S., Adeshina I., Mahmoudi R., Erfanifar E., Ajdari A. (2021). Selenium nanoparticle and selenomethionine as feed additives: effects on growth performance, hepatic enzymes' activity, mucosal immune parameters, liver histology, and appetite-related gene transcript in goldfish (*Carassius auratus*). Fish Physiol Biochem.

[bib57] Jiménez-Lamana J., Abad-Álvaro I., Bierla K., Laborda F., Szpunar J., Lobinski R. (2018). Detection and characterization of biogenic selenium nanoparticles in selenium-rich yeast by single particle ICPMS. J Anal At Spectrom.

[bib58] Kápolna E., Gergely V., Dernovics M., Illés A., Fodor P. (2007). Fate of selenium species in sesame seeds during simulated bakery process. J Food Eng.

[bib59] Kessi J., Ramuz M., Wehrli E., Spycher M., Bachofen R. (1999). Reduction of selenite and detoxification of elemental selenium by the phototrophic bacterium *Rhodospirillum rubrum*. Appl Environ Microbiol.

[bib60] Khalili M., Chamani M., Amanlou H., Nikkhah A., Sadeghi A.A., Dehkordi F.K., Rafiei M., Shirani V. (2020). The effect of feeding inorganic and organic selenium sources on the hematological blood parameters, reproduction and health of dairy cows in the transition period. Acta Sci Anim Sci.

[bib61] Khoso P.A., Pan T., Wan N., Yang Z., Liu C., Li S. (2017). Selenium deficiency induces autophagy in immune organs of chickens. Biol Trace Elem Res.

[bib62] Kieliszek M., Błażejak S. (2013). Selenium: significance, and outlook for supplementation. J Nutr.

[bib63] Kieliszek M., Błażejak S., Gientka I., Bzducha-Wróbel A. (2015). Accumulation and metabolism of selenium by yeast cells. Appl Microbiol Biotechnol.

[bib64] Kim Y.Y., Mahan D.C. (2001). Comparative effects of high dietary levels of organic and inorganic selenium on selenium toxicity of growing-finishing pigs. J Anim Sci.

[bib65] Kobayashi M., Suhara T., Baba Y., Kawasaki N.K., Higa J.K., Matsui T. (2018). Pathological roles of iron in cardiovascular disease. Curr Cancer Drug Targets.

[bib66] Koenig K.M., Buckley W.T., Shelford J.A. (1991). True absorption of selenium in dairy cows: stable isotope tracer methodology and effect of dietary copper. Can J Anim Sci.

[bib67] Koenig K.M., Beauchemin K.A. (2009). Supplementing selenium yeast to diets with adequate concentrations of selenium: selenium status, thyroid hormone concentrations and passive transfer of immunoglobulins in dairy cows and calves. Can J Anim Sci.

[bib68] Kohshahi A.J., Sourinejad I., Sarkheil M., Johari S.A. (2019). Dietary supplementation with curcumin and different selenium sources (nanoparticulate, organic, and inorganic selenium): influence on growth performance, body composition, immune responses,and glutathione peroxidase activity of rainbow trout (*Oncorhynchus mykiss*). Fish Physiol Biochem.

[bib69] Koller L.D., Exon J.H. (1986). The two faces of selenium – deficiency and toxicity – are similar in animals and man. Can J Vet Res.

[bib70] Kotrebai M., Birringer M., Tyson J.F., Block E., Uden P.C. (2000). Selenium speciation in enriched and natural samples by HPLC-ICP-MS and HPLC-ESI-MS with perfluorinated carboxylic acid ion-pairing agents. Analyst.

[bib71] Kumar A., Prasad K.S. (2021). Role of nano-selenium in health and environment. J Biotechnol.

[bib72] Lall S.P., Kaushik S.J. (2021). Nutrition and metabolism of minerals in fish. Animals.

[bib73] Larsen E.H., Łobinski R., Burger–Meÿer K., Hansen M., Ruzik L., Mazurowska L., Rasmussen P.H., Sloth J.J., Scholten O., Kik C. (2006). Uptake and speciation of selenium in garlic cultivated in soil amended with symbiotic fungi (mycorrhiza) and selenate. Anal Bioanal Chem.

[bib74] Liao X., Lu L., Li S., Liu S., Zhang L., Wang G., Li A., Luo X. (2012). Effects of selenium source and level on growth performance, tissue selenium concentrations, antioxidation, and immune functions of heat-stressed broilers. Biol Trace Elem Res.

[bib75] Lichtfouse E., Morin-Crini M., Bradu C., Boussouga Y., Aliaskari M., Schäfer A.I., Das S., Wilson L.D., Ike M., Inoue D., Kuroda M., Déon S., Fievet P., Crini G. (2022). Methods for selenium removal from contaminated waters: a review. Environ Chem Lett.

[bib76] Liu G., Zhao Y., Cao S., Luo X., Wang R., Zhang L., Lu L., Liao X. (2020). Relative bioavailability of selenium yeast for broilers fed a conventional corn–soybean meal diet. J Anim Physiol Anim Nutr.

[bib77] Longbaf Dezfouli M., Ghaedtaheri A., Keyvanshokooh S., Salati A.P., Mousavi S.M., Pasha-Zanoosi H. (2019). Combined or individual effects of dietary magnesium and selenium nanoparticles on growth performance, immunity, blood biochemistry and antioxidant status of Asian seabass (*Lates calcarifer*) reared in freshwater. Aquac Nutr.

[bib78] Lorentzen M., Maage A., Julshamn K. (1994). Effects of dietary selenite or selenomethionine on tissue selenium levels of Atlantic salmon (*Salmo salar*). Aquaculture.

[bib79] Lynch S., Horgan K., Walls D., White B. (2018). Selenised yeast sources differ in their capacity to protect porcine jejunal epithelial cells from cadmium-induced toxicity and oxidised DNA damage. Biometals.

[bib80] Malyugina S., Skalickova S., Skladanka J., Slama P., Horky P. (2021). Biogenic selenium nanoparticles in animal nutrition: a review. Agriculture.

[bib81] Marin-Guzman J., Mahan D.C., Chung Y.K., Pate J.L., Pope W.F. (1997). Effects of dietary selenium and vitamin E on boar performance and tissue responses, semen quality, and subsequent fertilization rates in mature gilts. J Anim Sci.

[bib82] Marin-Guzman J., Mahan D.C., Pate J.L. (2000). Effect of dietary selenium and vitamin E on spermatogenic development in boars. J Anim Sci.

[bib83] Marković R., Ćirić J., Starčević M., Šefer D., Baltić M. (2018). Effects of selenium source and level in diet on glutathione peroxidase activity, tissue selenium distribution, and growth performance in poultry. Anim Health Res Rev.

[bib84] Mavromichalis, I. Formulating pig diets: selenium toxicity, deficiency. Available online: https://www.wattagnet.com/articles/19843-formulating-pig-diets-selenium-toxicity-deficiency [accessed on 03.11.21].

[bib85] McLaren R.G., Clucas L.M. (2006). A field comparison of pasture selenium uptake from different forms of selenium fertiliser. NZJAR.

[bib86] Mehdi Y., Hornick J., Istasse L., Dufrasne I. (2013). Selenium in the environment, metabolism and involvement in body functions. Molecules.

[bib87] Mehdi Y., Dufrasne I. (2016). Selenium in cattle: a review. Molecules.

[bib88] Mester Z., Willie S., Yang L., Sturgeon R., Caruso J.A., Fernández M.L., Fodor P., Goldschmidt R.J., Goenaga-Infante H., Lobinski R., Maxwell P., McSheehy S., Polatajko A., Sadi B.B.M., Sanz-Medel A., Scriver C., Szpunar J., Wahlen R., Wolf W. (2006). Certification of a new selenized yeast reference material (SELM-1) for methionine, selenomethinone and total selenium content and its use in an intercomparison exercise for quantifying these analytes. Anal Bioanal Chem.

[bib89] Mocchegiani E., Romeo J., Malavolta M., Costarelli L., Giacconi R., Diaz L.E., Marcos A. (2013). Zinc: dietary intake and impact of supplementation on immune function in elderly. Age.

[bib90] Newberne P.M., Suphakarn V. (1983). Nutrition and cancer: a review, with emphasis on the role of vitamins C and E and selenium. Nutr Cancer.

[bib91] National Research Council (2012).

[bib92] National Research Council (2016).

[bib93] Ochsenkühn–Petropoulou M., Tsopelas F., Ruzik L., Bierła K., Szpunar J. (2006). Metallomics.

[bib94] Oropeza–Moe M., Wisløffb H., Bernhoftc A. (2015). Selenium deficiency associated porcine and human cardiomyopathies. J Trace Elem Med Biol.

[bib95] Pacitti D., Lawan M.M., Sweetman J., Martin S.A.M., Feldmann J., Secombes C.J. (2015). Selenium supplementation in fish: a combined chemical and biomolecular study to understand Sel-Plex assimilation and impact on selenoproteome expression in rainbow trout (*Oncorhynchus mykiss*). PLoS One.

[bib96] Paiva F.A., Saran Netto A., Corrêa L.B., Silva T.H., Guimarães I.C.S.B., Del Claro G.R., Cunha J.A., Zanetti M.A. (2019). Organic selenium supplementation increases muscle selenium content in growing lambs compared to inorganic source. Small Rumin Res.

[bib97] Patrick L. (2004). Selenium biochemistry and cancer: a review of the literature. Altern Med Rev.

[bib98] Penglase S., Hamre K., Ellingsen S. (2015). The selenium content of SEPP1 versus selenium requirements in vertebrates. PeerJ.

[bib99] Pilon-Smits E.A.H., Quinn C.F., Tapken W., Malagoli M., Schiavon M. (2009). Physiological functions of beneficial elements. Curr Opin Plant Biol.

[bib100] Pinotti L., Giromini C., Ottoboni M., Tretola M. (2019). Review: insects and former foodstuffs for upgrading food waste biomasses/streams to feed ingredients for farm animals. Animal.

[bib101] Pinotti L., Ottoboni M. (2021). Substrate as insect feed for bio–mass production. J Insects Food Feed.

[bib102] Puccinelli M., Malorgio F., Pezzarossa B. (2017). Selenium enrichment of horticultural crops. Molecules.

[bib103] Purschke B., Scheibelberger R., Axmann S., Adler A., Jager H. (2017). Impact of substrate contamination with mycotoxins, heavy metals and pesticides on growth performance and composition of black soldier fly larvae (*Hermetia illucens*) for use in the feed and food value chain. Food Addit Contam Part A.

[bib104] Radwan N.L., Eldin T.S., El-Zaiat A., Mostafa M.A. (2015). Effect of dietary nano-selenium supplementation on selenium content and oxidative stability in table eggs and productive performance of laying hens. Int J Poult.

[bib105] Rayman M.P. (2002). The argument for increasing selenium intake. Proc Nutr Soc.

[bib106] Rayman M.P. (2004). The use of high-selenium yeast to raise selenium status: how does it measure up?. Br J Nut.

[bib107] Ros G.H., van Rotterdam A.M.D., Bussink D.W., Bindraban P.S. (2016). Selenium fertilization strategies for bio-fortification of food: an agro-ecosystem approach. Plant Soil.

[bib108] Scheiber I., Dringen R., Mercer J.F.B. (2013). Copper: effects of deficiency and overload. Met Ions Life Sci.

[bib109] Schiavon M., Nardi S., dalla Vecchia F., Ertani A. (2020). Selenium biofortification in the 21st century: status and challenges for healthy human nutrition. Plant Soil.

[bib110] Schlegel P., Durosoy S., Jongbloed A.W. (2008).

[bib111] Schrauzer G.N. (2000). Anticarcinogenic effects of selenium. CMLS Cell Mol Life Sci.

[bib112] Schrauzer G.N. (2006). Selenium yeast: composition, quality, analysis, and safety. Pure Appl Chem.

[bib113] Schwarz K., Foltz C.M. (1957). Selenium as an integral part of factor 3 against dietary necrosis liver degeneration. J Am Chem Soc.

[bib114] Senthil Kumaran C., Sugapriya S., Manivannan N., Shekar C. (2015). Effect on the growth performance of broiler chickens by selenium nanoparticles supplementation. Nano Vis.

[bib115] Shi L., Xun W., Yue W., Zhang C., Ren Y., Qiang L., Wang Q., Shi L. (2011). Effect of elemental nano-selenium on feed digestibility, rumen fermentation, and purine derivatives in sheep. Fuel Energy Abstr.

[bib116] Shimizu A., Tobe R., Aono R., Inoue M., Hagita S., Kiriyama K., Toyotake Y., Ogawa T., Kurihara T., Goto K., Tejo Prakash N., Mihara H. (2021). Initial step of selenite reduction via thioredoxin for bacterial selenoprotein biosynthesis. Int J Mol Sci.

[bib117] Skalickova S., Milosavljevic V., Cihalova K., Horky P., Richtera L., Adam V. (2017). Selenium nanoparticles as a nutritional supplement. J Nutr.

[bib118] Slaweta R., Wasowiez W., Laskowska T. (1988). Selenium content, glutathione peroxidase activity and lipid peroxide level in fresh bull semen and its relationship to motility of spermatozoa after freezing and thawing. J Vet Med A Physiol Pathol Clin Med.

[bib119] Słovińska M., Jankowski J., Dietrich J., Karol H., Liszewska E., Glogowski J., Kozłowski K., Sartowska K., Ciereszko A. (2011). Effect of organic and inorganic forms of selenium in diets on Turkey semen quality. Poult Sci.

[bib120] Spranghers T., Ottoboni M., Klootwijk C., Ovyn A., Deboosere S., De Meulenaer B., Michiels J., Eeckhout M., De Clercq P., De Smet S. (2017). Nutritional composition of black soldier fly (*Hermetia illucens*) prepupae reared on different organic waste substrates. J Sci Food Agric.

[bib121] Star L., Arsiwalla T., Molist F., Leushuis R., Dalim M., Paul A. (2020). Gradual provision of live black soldier fly (*Hermetia illucens*) larvae to older laying hens: effect on production performance, egg quality, feather condition and behavior. Animals.

[bib122] Surai P.F. (2006).

[bib151] Surai P.F., Fisinin V.I. (2015). Selenium in pig nutrition and reproduction: boars and semen quality-a review. Asian-Australas J Anim Sci.

[bib123] Surai P.F., Fisinin V.I., Karadas F. (2016). Antioxidant systems in chick embryo development. Part 1. Vitamin E, carotenoids and selenium. Anim Nutr.

[bib124] Surai P., Kochish I.I., Fisinin V.I., Juniper D.T. (2019). Revisiting oxidative stress and the use of organic selenium in dairy cow nutrition. Animals.

[bib125] Suttle N. (2010).

[bib126] Tan L.C., Nancharaiah Y.V., van Hullebusch E.D., Lens P.N.L. (2016). Selenium: environmental significance, pollution, and biological treatment technologies. Biotechnol Adv.

[bib127] Tan X., Yang H., Wang M., Yi Z., Ji F., Li J., Yin Y. (2020). Amino acid digestibility in housefly and black soldier fly prepupae by growing pigs. Anim Feed Sci Technol.

[bib128] Tarze A., Dauplais M., Grigoras I., Lazard M., Ha-Duong N.T., Barbier F., Blanquet S., Plateau P. (2007). Extracellular production of hydrogen selenide accounts for thiol-assisted toxicity of selenite against *Saccharomyces cerevisiae*. J Biol Chem.

[bib129] Thompson J.N., Scott M.L. (1969). Role of selenium in the nutrition of the chick. J Nutr.

[bib130] Tian J.Z., Yun M.S., Ju W.S., Long H.F., Kim J.H., Kil D.Y., Chang J.S., Cho S.B., Kim Y.Y., Han I.K. (2006). Effects of dietary selenium supplementation on growth performance, selenium retention in tissues and nutrient digestibility in growing-finishing pigs. Asian-Aust J Anim Sci.

[bib131] Tugarova A.V., Vetchinkina E.P., Loshchinina E.A., Burov A.M., Nikitina V.E., Kamnev A.A. (2014). Reduction of selenite by azospirillum brasilense with the formation of selenium nanoparticles. Microb Ecol.

[bib132] Underwood E.J., Suttle N.F. (1999).

[bib133] Vacchina V., Foix D., Menta M., Martinez H., Séby F. (2021). Optimization of elemental selenium (Se(0)) determination in yeasts by anion-exchange HPLC-ICP-MS. Anal Bioanal Chem.

[bib134] von Holst C., Robouch P., Bellorini S., Gonzálezde la Huebra M.S., Ezerskis Z. (2016). A review of the work of the EU Reference Laboratory supporting the authorisation process of feed additives in the EU. Food Addit Contam Part A.

[bib135] Wadhwani S.A., Shedbalkar U.U., Singh R., Chopade B.A. (2016). Biogenic selenium nanoparticles: current status and future prospects. Appl Microbiol Biotechnol.

[bib136] Wang Y.-B., Xu B.-H. (2008). Effect of different selenium source (sodium selenite and selenium yeast) on broiler chickens. Anim Feed Sci Technol.

[bib137] Wang L., Zhang X., Wu L., Liu Q., Zhang D., Yin J. (2018). Expression of selenoprotein genes in muscle is crucial for the growth of rainbow trout (*Oncorhynchus mykiss*) fed diets supplemented with selenium yeast. Aquaculture.

[bib138] Wastney M.E., Combs G.F., Canfield W.K., Taylor P.R., Patterson K.Y., Hill A.D., Moler J.E., Patterson B.H. (2011). A human model of selenium that integrates metabolism from selenite and selenomethionine. J Nutr.

[bib139] Whanger P.D. (2002). Selenocompounds in plants and animals and their biological significance. J Am Coll Nutr.

[bib140] White P.J., Bowen H.C., Parmaguru P., Fritz M., Spracklen W.P., Spiby R.E., Meacham M.C., Mead A., Harriman M., Trueman L.J., Smith B.M., Thomas B., Broadley M.R. (2004). Interactions between selenium and sulphur nutrition in *Arabidopsis thaliana*. J Exp Bot.

[bib141] Wilkins J.F., Kilgour R.J. (1982). Production responses to selenium in northern New South Wales. 1. Infertility in ewes and associated production. Aust J Exp Agric Anim Husb.

[bib142] Xiong L., Li B., Yang Y. (2018). Effects of foliar selenite on the nutrient components of turnip (*Brassica rapa* var. rapa Linn.). Front Chem.

[bib143] Xun W., Shi L., Yue W., Zhang C., Ren Y., Qiang L. (2012). Effect of high-dose nano-selenium and selenium–yeast on feed digestibility, rumen fermentation, and purine derivatives in sheep. Biol Trace Elem Res.

[bib144] Yan J., Chen X., Zhu T., Zhang Z., Fan J. (2021). Effects of selenium fertilizer application on yield and selenium accumulation characteristics of different Japonica rice varieties. Sustainability.

[bib145] Yang Z., Liu C., Liu C., Teng X., Li S. (2016). Selenium deficiency mainly influences antioxidant selenoproteins expression in broiler immune organs. Biol Trace Elem Res.

[bib146] Ye Y., Qu J., Pu Y., Rao S., Xu F., Wu C. (2020). Selenium biofortification of crop food by beneficial microorganisms. J Fungi.

[bib147] Yuan Z., Long W., Liang T., Zhu M., Zhu A., Luo X. (2022). Effect of foliar spraying of organic and inorganic selenium fertilizers during different growth stages on selenium accumulation and speciation in rice. Plant Soil.

[bib148] Zhang K., Zhao Q., Zhan T., Han Y., Tang C., Zhang J. (2020). Effect of different selenium sources on growth performance, tissue selenium content, meat quality, and selenoprotein gene expression in finishing pigs. Biol Trace Elem Res.

[bib149] Zhou X., Yang J., Kronzucker H.J., Shi W. (2020). Selenium biofortification and interaction with other elements in plants: a review. Front Plant Sci.

[bib150] Zoidis E., Seremelis I., Kontopoulos N., Dazenis G.P. (2018). Selenium-dependent antioxidant enzymes: actions and properties of selenoproteins. Antioxidants.

